# Time to STEP UP: methods and findings from the development of guidance to help researchers design inclusive clinical trials

**DOI:** 10.1186/s12874-024-02342-y

**Published:** 2024-10-02

**Authors:** K. Biggs, K. Hullock, C. Dix, JA. Lane, H. Green, S. Treweek, F. Shiely, V. Shepherd, A. Willis, T. Isaacs, C. Cooper

**Affiliations:** 1 Clinical Trials Research Unit, Division of Population Health, University of Sheffield, Sheffield, UK; 2 Bristol Medical School, Bristol Trials Centre, University of Bristol, Bristol, UK; 3 Health Services Research Unit, University of Aberdeen, Aberdeen, UK; 4 COUCH Health, Manchester, UK; 5 HRB Clinical Research Facility and School of Public Health, University College Cork, Cork, Ireland; 6 Centre for Trials Research, Cardiff University, Cardiff, UK; 7 IOE, UCL’s Faculty of Education and Society, University College London, London, UK

**Keywords:** Inclusion, Diversity, Under-served groups, Randomised controlled trials, Research design

## Abstract

**Background:**

It is important to design clinical trials to include all those who may benefit from the intervention being tested. Several frameworks have been developed to help researchers think about the barriers to inclusion of particular under-served groups when designing a trial, but there is a lack of practical guidance on how to implement these frameworks. This paper describes the ACCESS project, the findings from each phase of the project and the guidance we developed (STEP UP) on how to design more inclusive trials.

**Methods:**

Development of the STEP UP guidance had five phases: (1) Scoping literature review, (2) ‘roundtable’ discussion meetings, (3) redesign of trials, (4) interviews and (5) guidance document development, with input from public contributors and the ACCESS team.

**Results:**

Over 40 experts contributed to the ACCESS project—patients and the public, clinicians, NHS research staff, trialists and other academics. The scoping review identified several strategies being used to improve inclusion, mostly around recruitment settings, but there was little evaluation of these strategies. The ‘roundtable’ discussions identified additional strategies being used across the UK and Ireland to improve inclusion, which were grouped into: Communication, Community engagement, Recruitment sites, Patient information, Flexibility, Recruitment settings, Consent process, Monitoring, Training for researchers and Incentives. These strategies were used to redesign three existing trials by applying one of the three INCLUDE frameworks (ethnicity, socioeconomic disadvantage, impaired capacity to consent) to one trial each, to produce the key recommendations for the guidance. Issues around implementation were explored in stakeholder interviews and key facilitators were identified: funders requesting information on inclusion, having the time and funding to implement strategies, dedicated staff, flexibility in trial protocols, and considering inclusion of under-served groups at the design stages. The STEP UP guidance is freely available at http://step-up-clinical-trials.co.uk.

**Conclusion:**

Researchers should consider inclusivity to shape initial trial design decisions. Trial teams and funders need to ensure that trials are given both the resources and time needed to implement the STEP UP guidance and increase the opportunities to recruit a diverse population.

**Supplementary Information:**

The online version contains supplementary material available at 10.1186/s12874-024-02342-y.

## Plain Language Summary

Randomised clinical trials compare one or more treatments to another to see which ones work best. Trials don’t always include people or groups who might benefit from the results: those excluded are sometimes called ‘under- served groups’. Recent work has shone a light on this and now researchers are being asked by the public, trial funders and others to design their research so that under-served groups are more able to take part.

We worked on a project to find out how to make sure everyone can be part of clinical trials. We looked at published work and held five online meetings with researchers, doctors, and patients to see what was being done already, and to think of other things that could help under-served groups take part in trials. Three groups of people, including scientists, patients, doctors and other NHS workers then  used this information to redesign three older trials using some existing inclusivity frameworks to think through the barriers for under-served groups in these trials. The three groups then talked through these trials at a 2-hour meeting, suggesting changes to the original trial plan, and discussed whether the suggestions were practical and useful. From this we came up with recommendations for how to design trials so that they have fewer barriers for under-served groups.

We interviewed people to find out the best way to put these things into practice and talk through any practical issues. Using all of this information: the recommendations and what came out of the interviews, the study team created some guidance – ‘STEP UP (Strategies for Trialists to promote Equal Participation in clinical trials for Under-served Populations)’ – for people working in trials.

**Supplementary Information:**

The online version contains supplementary material available at 10.1186/s12874-024-02342-y.

## Background

Research shows that participants in clinical trials are often unrepresentative of the populations that could benefit from the treatments being investigated. To help to address this, the National Institute for Health and Care Research (NIHR) INCLUDE project [1, 2] was initiated in 2017 to look at under-representation in clinical trials. It identified a range of under-served groups, which can vary across the types of studies, disease or condition being studied. This has  prompted funders and trialists to make efforts to improve the recruitment and retention of under-served groups by thinking about the way trials are designed. There has been extensive work around improving recruitment and retention to trials which may be useful in improving the recruitment and retention of under-served groups, but they did not focus on under-served populations [3–5]. To help researchers design more inclusive trials, there are currently three INCLUDE Frameworks [6–8], each aiming to support researchers to think carefully about their trials with ethnicity, people with impaired capacity to consent, and people experiencing socioeconomic disadvantage in mind. The Frameworks guide discussions around which groups of people the trial results should apply to, and how the design of the trial and the intervention might make it more difficult for any group to take part. The INCLUDE frameworks help researchers (alongside clinicians, patients and the public) to consider how elements of the trial might facilitate diverse recruitment, or create barriers and make amendments to their design accordingly. 

## The ACCESS project

The Medical Research Council-NIHR-Trial Methodology Research Partnership (MRC-NIHR-TMRP) Inclusivity Working Group identified that guidance on how to overcome some of these barriers to inclusion was needed. A collaborator group was formed through the TMRP working group and the UK Clinical Research Collaboration (UKCRC) network and we set up the ACCESS project (‘*A collaborative study to identify the activities needed to improve representation of under-served groups in trials and understand their implementation’* – https://www.sheffield.ac.uk/ctru/completed-trials/access) funded through the NIHR CTU trial efficiency funding programme. Although there was no patient or public involvement (PPI) contributor in the ACCESS team we sought PPI throughout the project, including the development of the guidance. The ACCESS project aimed to identify how trials can be designed to make them more accessible to under-served groups.

## The STEP UP guidance

This paper describes the process used to develop the STEP UP (Strategies for Trialists to promote Equal participation  in clinical trials for Under-served Populations) guidance for trialists (http://step-up-clinical-trials.co.uk). The guidance has been developed to help trial teams design trials that are more accessible to a wider range of people, including under-served groups [1]. It can be used in conjunction with other resources and guidance that aims to improve inclusion in trials such as the INCLUDE frameworks [6–8], or Equality Impact Assessments [9].

## Developing the STEP UP guidance

**Fig. 1:**
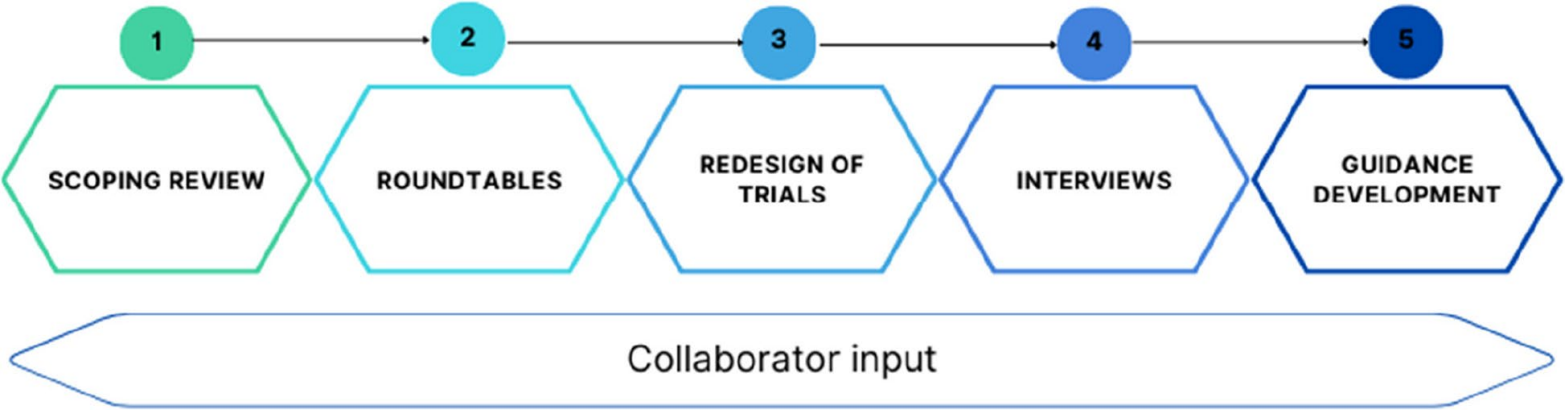
ACCESS project phases

Five phases made up the ACCESS project with input from the ACCESS collaborators throughout (Fig. 1); a full protocol can be found on the project website. Ethical approval for phases 2–4 was given by the School of Health and Related Research (ScHARR) Research Ethics Committee (REC) in January 2022 (Reference: 043869). Participants in all stages of the ACCESS project were invited through the ACCESS team’s contacts and networks, emails to distribution lists for trialists, and advertisements through the NIHR’s People in Research website (https://www.peopleinresearch.org). PPI members and interviewees were reimbursed for their time in line with the NIHR payment guidance [10]. Contributors at each phase are detailed in appendices (Appendices, Table 1).

## Phase 1: Scoping review (October 2021-January 2022)

### Methods

We conducted a scoping review of methodological research studies from the UK and Ireland to identify effective activities to improve representation of under-served groups in trials [11]. We focused on minoritised ethnic groups, socioeconomically disadvantaged groups, older people, and those with impaired capacity to consent, published between 2000–2021. These groups were chosen in line with the existing INCLUDE frameworks [6–8] and current work in the area [12]. Systematic searches were conducted in November 2021 using the PubMed database. Data were independently extracted by two authors (CD and KB) and narratively synthesised. The methods and findings from the scoping review have been previously reported in full [11].

### Findings

#### Strategies

We identified seven papers discussing strategies for improving inclusion [13–19]. We grouped the strategies they discussed into nine domains based on a previous review of strategies (without evaluation) in this area [20]: Recruitment sites; Recruitment settings; Community engagement; Patient documentation; Communication between study team and participants; Flexibility; Incentives; Inclusion criteria; Consent process. The most common domain mentioned across the papers was the recruitment setting, with a broad range of recruitment venues reported.

#### Evaluation

Although some activities appeared promising in engaging under-served populations, there was little evaluation of the effectiveness of these activities on recruitment or retention of under-served groups. Formal evaluation showed a monetary incentive mentioned in an invitation letter improved positive responses overall, but not for older people or people experiencing socioeconomic disadvantage [13]. Comparisons of recruitment pathways indicated that letters from GPs were the most successful method in recruiting older populations [15, 21], but other methods might be better for recruiting socioeconomically or ethnically diverse participants [15].

Recruitment via community organisations, and via snowball sampling were reported as successful by the authors for recruiting South Asian participants [16], and talks in community venues for recruiting more diverse participants [15]. Using mixed methods, Jayes and Palmer [18] found that the Consent Support Tool (CST) successfully identifies the appropriate information to give the participant based on their aphasia and can be used in the trial consent process. Only two trials reported retention data [17, 21], with retention rates being 85% or higher in both. Kolovou et al. [17] used strategies around communication, flexibility in follow-up visits and incentives to maximise retention, and Forster et al. [14] reported that their £100 incentive may have helped with retention rather than recruitment as it was mentioned after recruitment.

### ACCESS team input

A collaborator meeting was held to discuss the findings of the scoping review, to plan the format of the roundtable meetings and to identify possible contacts and networks for participants. Suggestions were made based on knowledge of other projects being conducted with the aim of improving inclusion in trials across the UK and Ireland.

## Phase 2: Roundtable Discussions (3rd, 7th, 18th, 25th and 28th March 2022)

### Methods

We had five online ‘roundtables’ with a total of 30 attendees (4–8 attendees per meeting) including trialists, recruiting staff, clinicians, and PPI contributors. The meetings were approximately two hours long and started with a presentation of the scoping review findings, we then facilitated an open discussion guided by the domains from the review. Activities discussed by the attendees were added to a ‘Jamboard’ during the meeting (Appendices, Fig. 1), and all participants were given the opportunity to comment on these during the meeting. The ‘Jamboards’ and notes taken by CD and KB during the meeting were combined to produce a list of suggested strategies (Table 1).

**Table 1:** Activities to support more inclusive trials identified by participants attending the roundtables

Domain	Suggested strategies
Communication between study team and participants	• Use simple language, and check the reading age of text to ensure accessibility of information; use less ‘academic’ language. • Communication should be tailored to the participants’ needs, for example ‘Easy Read’ leaflets for people with learning disabilities, or using translations or interpreters for those with limited English proficiency. • A person-specific approach to communication is required. • Consider a range of communication methods to engage participants, for example, the use of graphics. • Use social media and local radio to reach a larger audience for recruitment. • Use video and audio versions in several languages and build-in these costs from the beginning of the research as they're expensive. • Work with community groups to know which languages are required to be inclusive of target populations. • Diversity within clinical / research team can improve trust in researchers and research; this can help with communication due to a shared cultural understanding. • Sensitive information should be shared by a trusted person. • Researchers should maintain communication with participants throughout the process of the research. • Where people have not been chosen to participate or are ineligible, this should be fully explained to them.
Community engagement	• Researchers should build lasting, bi-directional relationships with communities. • Researchers should take the time to learn who the trusted voices are within the communities. • Key members of community groups can act as research champions and talk on behalf of the research team within that community. • There should be wider community engagement in clinical trials generally – more education around what clinical trials are will reduce scepticism and build more trust. • Researchers can connect with charities linked to specific under-served groups and have a pre-established relationship with potential participants. • Researchers should explicitly state that the research team is looking for specific under-served groups due to historical lack of representation. • Enlist former participants to act as research champions to recruit other participants. • Promote an environment of open discussion during the recruitment process and during PPI to tackle the idea of researcher hierarchy, and reduce power imbalances. • Greater presence of health champions in GPs could help with education around clinical trials and recruitment to trials. • Researchers should keep participants updated on the outcome of studies they have been involved in to foster greater trust and sense of involvement rather than feeling neglected once they have finished contributing. • Connect with Patient Participation Groups in General Practice as well as PPI groups. • Include patients and communities in dissemination of results and ensure lay summaries are done correctly. • Plan accessible dissemination events, for example hold them in the evening and go to community venues. • Visit and recruit from places where under-served groups are comfortable. • Involve more than one PPI person in your project, more people will provide a range of views and may highlight barriers to your research not previously considered. • PPI should be more diverse. • Diverse PPI should be involved from the start of the project, to the sharing of the results.
Recruitment sites	• Locations should be more inclusive of participants based in rural areas and accessible to those who have mobility issues.
Delivery of patient information	• Use visual and audio communication methods – videos, graphics, audio recordings. • Translation is not always useful if documentation is still complex and lengthy. More useful to have narrated videos in different languages (and in more everyday language as often translations are very formal). • Use information layering, with shorter documents explaining what is important to the patient in addition to the main patient information sheet. • Consider taking consent in stages rather than overburdening participants with excessive information all at once. • Patient documentation should be co-produced to ensure the information provided is understandable and appropriate.
Flexibility	• Recognise individual participant needs, and provide options for people to take part where possible. • Recognise and tackle patient burden. For example, participants in rural areas who already struggle to access healthcare services may need additional support to take part. • Reduce/remove unnecessary outcome measurements; focus on key outcomes if participants find all of the outcomes difficult to complete. • There should be more flexibility with English language as an eligibility criteria. • Researchers should be understanding of participants’ situations and not be punitive of non-attendance or lateness but recognise and acknowledge participants’ situations. • Be flexible in where and when to meet participants (for example, via community outreach) rather than trying to fit them into a pre-designed format. • Include costs for computer equipment and internet costs for any remote activity to enable participants who may not have access to these to participate.
Recruitment settings	• Use GP referrals to recruit participants as most people are registered with a GP. • Build relationships with non-NHS organisations and compile lists of contacts, such as care homes to enable quicker recruitment of target populations. • Consider research networks to help with recruitment, for example, the NIHR ENRICH project network to help with research in care homes. • Target broader areas rather than limiting recruitment to research hubs or large hospitals. • Use libraries in communities for recruitment purposes to reach more rural areas.
Consent process	• Simplify the consent process to improve understanding and accessibility; check the process and documentation with people from under-served groups. • Participants’ understanding should be checked frequently during consent. • Consider providing, and budgeting for, scribes for participants who have literacy or writing limitations. • E-consent should be offered as an option to reduce patient burden but this should not be the only option, as it might exclude those who are not computer literate or do not have access to technology.
Monitoring and Evaluation	• Report participant demographics during the trial so the composition of the trial population can be monitored. • Reporting of retention by under-served group to determine differences in attrition. • It was noted that the efficiency of trials needs to be reconsidered as long-term efficiency rather than just hitting recruitment targets by a certain date.
Training for researchers	• Training should be provided to researchers about how best to engage with PPI members and respect that not all PPI is the same. • Cultural awareness training should be given to researchers in order to be more inclusive for participants from underrepresented groups. • More diversity and training is needed within ethics committees to encourage the use of these strategies in trials.
Incentives	• Participants generally felt incentives are not that useful. • People thought they should be paid for the time they put into the trial and that fair remuneration is not an incentive. • Adequate payment is essential, and payment systems should not take too long to pay people. Most people would like immediate reimbursement.

### Findings

While roundtable discussions identified more activities aiming to promote inclusivity in trials compared to the scoping review, a gap remains in evaluating their effectiveness. Barriers to evaluation highlighted by participants were the tight deadlines in research, and the importance of recruitment, where researchers want to do everything that they can to improve recruitment, so all available strategies are adopted, implemented and usually not tested.

Several common items were discussed across all meetings. Simple language, clinician/researcher attitudes and communication with participants, and community engagement were considered important throughout. Table 1 summaries the strategies mentioned by participants at the five roundtables, ordered according to the most commonly discussed themes across the roundtables.

The importance of intersectionality was highlighted in all roundtables, where several PPI contributors considered themselves to be part of more than one under-served group. There were discussions around socioeconomic disadvantage being linked to other under-served groups, such as ethnic minorities, stigmatised populations, people with disabilities and/or mental health conditions, people in alternative residential circumstances, and that the intersection of these identities can result in health inequalities. This can create additional barriers to participating in trials, and highlighted the need for trialists to consider more than one under-served group in their trials in order to be inclusive.

Attendees had different preferences in relation to several elements of trial design. For example, in relation to the methods used for advertising the trial, some people preferred finding out face-to-face via their healthcare provider, others preferred letters from the GP, or advocated for using social media. Different consent and data collection methods were suggested for different groups. For example, some people suggested that older people might prefer a visit, and talking through the documents to remote forms of consent, but that remote data collection might be preferred to reduce clinic visits for those with less time. It is also important to note that members of an under-served group are not homogenous and can have different preferences, and that more than one method is needed across the trial stages to avoid excluding people.

Roundtable attendees also highlighted the importance of other organisations’ support in making trials more accessible. It was noted that research ethics committees (RECs) have a role to play in requesting that researchers explain how they will recruit a diverse population in the same way that they require details on other parts of the recruitment process, and more recently PPI. There appears to be variation in RECs and there was a suggestion that REC members may benefit from training around inclusion. Funding and timelines are likely to be increased when aiming for diverse populations, and pressure on recruitment timelines was reported as a barrier for researchers in doing these activities, therefore support from funders is necessary.

### ACCESS team member input

ACCESS team members discussed the inclusion activities suggested at the roundtables and were reassured that a lot of the same suggestions were coming up across the five roundtables and from different stakeholders. ACCESS team members suggested looking at the demographics of the meeting attendees to see if suggestions varied by the different demographic groups. The main difference in content was the under-served groups discussed at the meeting, but the suggested implementation strategies did not differ (see appendices, Fig. 1), e.g., engagement with communities for research processes and clinical trial education, using various recruitment settings, using simple and inclusive language, appropriately trained staff and making sure PPI is appropriate and includes people from under-served groups. The ACCESS team discussed using different recruitment settings in a trial, which led to discussion around the focus of the research question, as this often determines the setting that the population can be recruited from as community-based recruitment may be less successful for studies focussing on a particular clinical population. For example, surgical trials can often only recruit those on the waiting list for that particular surgery, and cannot recruit via community groups. It is for funders and researchers to consider the scope of the research question carefully, and how this might exclude people with poor healthcare access. ACCESS team members discussed the existing pressures to recruit, which often override efforts to recruit a more diverse sample and agreed that efficiency of recruitment should be considered in light of having a more inclusive sample.

ACCESS team members decided the types of trials to be selected for the next phase of the project. To ensure a broad range of contexts were considered, it was decided that one should involve primary care as this came up a lot in the roundtable meetings, one should involve a care home as there are additional considerations around older people and mental capacity, and one should be a drug trial as that may involve additional safety and regulatory issues.

## Phase 3: Redesign meetings (June 2022-October 2022)

### Methods

We had three online meetings including several stakeholders (see Table 1 in appendices) to theoretically redesign three completed trials that ran in the UK and were funded by the NIHR. Three trials were chosen (Table 2) to cover different types of intervention and conditions (drug trial in diabetes, therapy for depression, and a care home occupational therapy trial for stroke) which enabled focus on different under-served groups: Ethnic minority communities, older people, socio-economically disadvantaged groups, and people with impaired capacity to consent. Prior to each meeting the study team went through an INCLUDE framework relevant to the trial, making suggestions based on the scoping review and roundtable findings, and these were reviewed by ACCESS team members. These suggestions were organised around four key questions in the INCLUDE frameworks, and elements of trial design, and circulated to attendees one week before the meeting to review in advance if they wished. Due to the time allocated for the meeting, and to reduce the burden for attendees, we did not share the patient information materials and so did not discuss these elements in detail during the redesign meetings. We had limited information on some elements of the trials, such as PPI involvement, but these were still discussed, and suggestions made where appropriate.

**Table 2:** PICO for trials and INCLUDE framework used for theoretical redesign

	Stroke trial Meeting date: 16/06/2022	Depression trial Meeting date: 02/09/2022	Diabetes trial Meeting date: 03/10/2022
Population; Recruitment setting	Care home residents with confirmed or suspected stroke; Care homes.	Adults with depression who scored ≥ 10 on the Patient Health Questionnaire-9 (PHQ-9); Primary care/General Practices (GPs)	Patients with Diabetic Peripheral Neuropathic Pain (DPNP); Secondary care/hospitals.
Intervention	Occupation therapy (OT) package was delivered to residents and care home staff.	Two intervention groups: two types of online Cognitive Behavioural Therapy.	Six sequences consisting of 3 drug pathways.
Comparator	Usual care.	Usual care by their GP.	Placebo.
Outcome	Barthel Index score (assesses dependency).	Depression severity and symptomatology as measured by a validated self-report measure (PHQ-9).	7-day average 24-h pain (evaluated at patient level) on an 11 point Numeric rating Scale (0 = no pain and 10 = worst pain imaginable).
INCLUDE framework used	Impaired capacity to consent framework [8].	Socio-economic disadvantage framework [7].	Ethnicity framework [6].

Each meeting started with a 10-min presentation on the ACCESS findings so far and the INCLUDE framework key questions, and then went through the elements of the trial. We presented the ACCESS team suggestions, allowing participants to discuss freely and make additional suggestions. KB presented the findings and suggestions, and chaired the meetings and a research assistant took notes (CD or KH). Discussion started with thinking about the population that should be included in the design and considered the eligibility criteria, which helped to think about the other elements of trial design as per the INCLUDE frameworks.

## Trials and frameworks

### Findings

Table 3 details the suggestions made by the ACCESS team based on previous phases of the project, and comments and suggestions from the stakeholders at each meeting. There were several common elements redesigned in each meeting, and some similar considerations, even though the focus was on different populations, under-served groups and included stakeholders with different experience.

**Table 3:** Redesign recommendations by trial element and by trial

**Area for discussion**	**Trial **	**ACCESS team suggestions based on phases 1 & 2**	**Redesign meeting comments**
Trial population	Stroke	Broadened the population to include community-based participants due to disparity in terms of ethnicity in care homes.	• If the intervention can be delivered in a care home (or in patient’s homes), the research should be there too. • Monitor the proportion of different ethnic groups recruited and retained throughout the trial. • An Equality Impact Assessment, or INCLUDE framework should be completed when developing the research question.
	Depression	(Diverse) PPI in design of the trial. Use video participant information to ensure engagement across groups.	• Diagnosis of depression can be difficult, especially for those not engaging in services - invite people with other conditions via GP. • Older people may not find the trial’s online intervention as acceptable - they may lack access or think they are not capable which could be covered in recruitment discussions. • Consider reframing the exclusion criteria around psychotic symptoms as there was suggestion of over diagnosis in some cultures, meaning more people from those groups might be disproportionately excluded.
	Diabetes	South Asian population disproportionally affected by diabetes, so this group should be considered in the design. Lots of exclusions for the trial – need to consider if all are necessary.	• Link to South Asian communities and include members of the community in the in design. • Other under-served groups need to be considered: Other ethnic minority groups; prisoners, rural populations. • Exclusion criteria considered too strict, but mostly clinically relevant or for safety. Eligibility criteria should be operationalised so recruiting staff don’t overly exclude some people, e.g. level of English, substance misuse, serious mental illness – these may be justified but perhaps too binary.
Recruitment settings	Stroke	Broadened to community based participants living at home.	• Care homes with different types of funding for residents’ care, such as self-funded or local authority funded, should be included to ensure a more diverse sample. • Do not just go to homes that are ‘research ready’ as they may not be as diverse. • Funders should ask where teams aim to recruit from at early stages.
	Depression	Addition of a few more recruitment pathways, particularly through communities.	• As a depression measure was used for screening to the trial, groups that will not have a depression diagnosis could be invited, though this may increase the work for those screening. • Include social prescribers in recruitment and research. • Train lay advisors in the community, for GPs. • Target GPs in diverse areas.
	Diabetes	South Asian population is disproportionally affected by diabetes.	• Stakeholder engagement is needed. • Community groups can act as gatekeepers for recruitment and advocates for the research.
Consent & communications	Stroke	Cultural awareness training for occupational therapists. Employ diverse therapists to deliver the intervention	• Employ diverse therapists to deliver the intervention; have a diverse research team.
	Depression	Only delivered in English; no cultural adaptations. Fidelity assessment would be needed for any adaptations.	• It would be better if the intervention could be delivered in another language - therapists may be able to do this with interpreters for face-to-face therapy, but would need a lot more work to develop several versions of online therapy. • Where translated versions are possible, there is a question about whether the same intervention is then tested in a trial.
	Diabetes	The trial had a complex schedule and drug combinations which need to be well explained. Risk of the medication not working and washout periods lead to poor attrition, and so should be well explained.	• A video explaining the dosing schedules might be helpful to aid understanding. • Drugs could be delivered remotely. • If the safety tests could be delivered remotely they could be done this way to reduce clinic visits.
Outcomes and follow-up	Stroke	Allows proxy completion by staff, this could be widened to friends and family. There should be a process for what happens if participant loses capacity during the trial.	• Choose outcomes that have been tested across groups of people, and can be completed by proxy. • Clinical measures require less input from participants, and can be collected from patients unable to complete forms. • Measure retention across groups.
	Depression	Funding for travel should be included. Flexibility in timing (e.g., out of hours) for study visits. Use alternative methods of follow-up (e.g. online, telephone, postal, clinic visits, home visits).	• Childcare costs and time off work for participant visits should be included, and where possible given up front – reimbursement may not help if people don’t have the money to start with. • Additional consideration is needed for those who live rurally or not near a large teaching hospital. • Researchers should show understanding of why people may miss appointments, not ‘tell people off’.
	Diabetes		
Analysis and dissemination	Stroke	Sub-group analysis could be conducted for different groups – will not be powered but may provide useful information. Dissemination must be wider than academic publication and include community groups.	• Sub-group analysis on frailty instead of ageing as health inequalities shape ageing.
	Depression		• Monitor drop out by underserved group.
	Diabetes		• Ask trusts and charities to circulate results. • Present results in a one page, eye catching report. • Ask communities how to disseminate. • Use videos to disseminate results – but they need to be short for social media. • Present the results in different languages.

### ACCESS team member input

There was at least one member of the ACCESS team at each of the redesign meetings and all recommendations for redesigning the trial were presented to the ACCESS team members at a further meeting. We discussed potential implementation issues, and how some of the recommendations could be operationalised in the guidance.

In relation to who should be included in the interviews to discuss implementation issues, we agreed to include trialists that had not been included so far, and people who were involved in the roundtables who had relevant trial experience. ACCESS team members discussed the need to focus on more than one under-served group in the discussions so that the guidance could cover wider aspects of trial design.

## Phase 4: Interviews and ACCESS team member meeting on implementation (December 2022-March 2023)

### Methods

To explore the implementation of inclusive trial designs, we conducted 15 interviews with CTU staff, clinical trialists, community experts and researchers with experience of including under-served groups in heath research. We then held an ACCESS team member meeting to discuss issues around implementing these activities.

We explored interviewee experiences in implementing activities aimed at improving representation of underserved groups, determined if they were successful, and we focused on the facilitators and barriers to their implementation.

### Findings

**Fig. 2:**
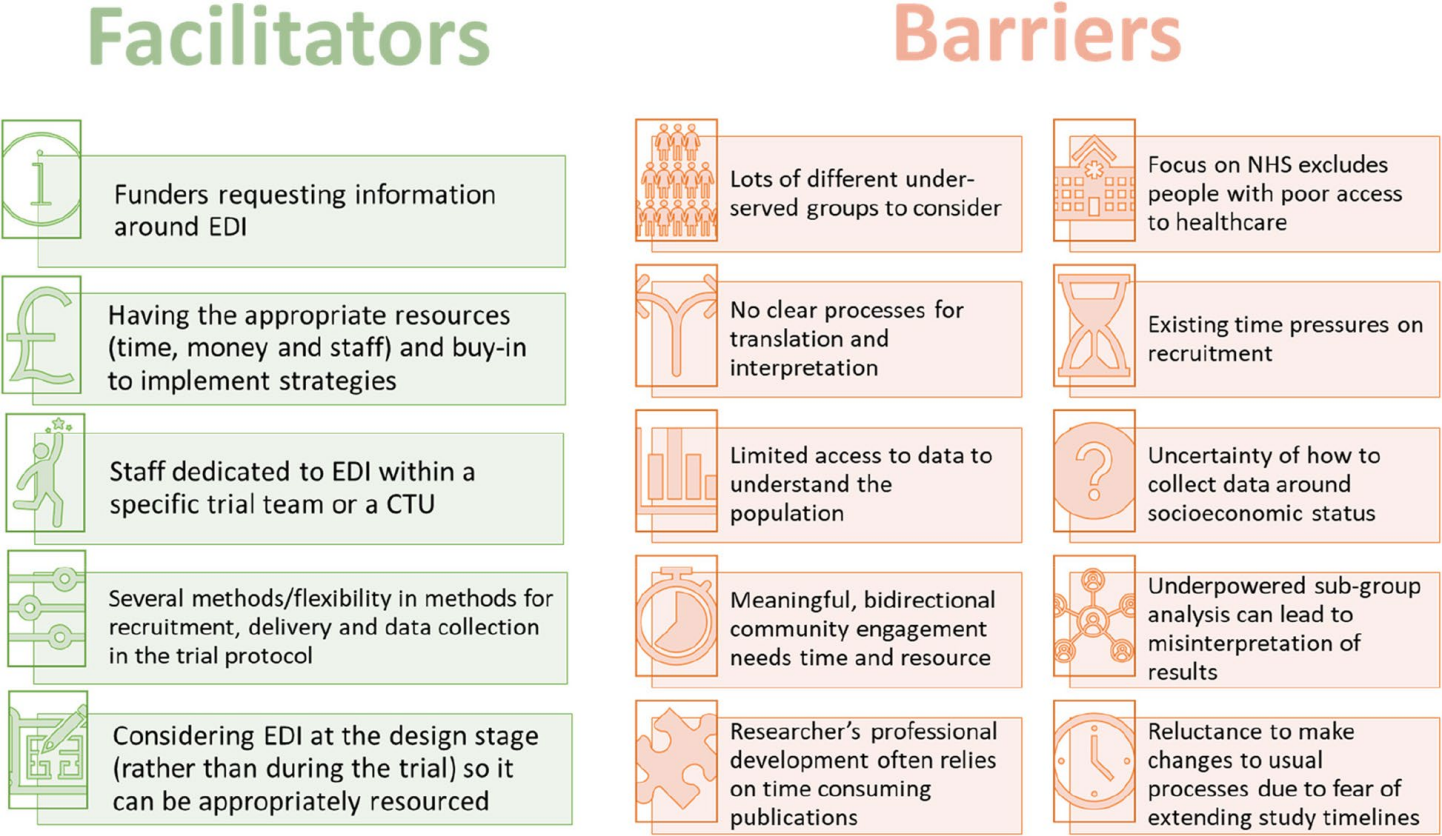
Facilitators and barriers to implementing strategies to improve inclusion identified in interviews

The key implementation issues identified in the interviews and the ACCESS team member meeting (and throughout the ACCESS work-packages) are presented in Fig. 2. During each ACCESS work package, the project team took notes of the barriers and facilitators as they were discussed in relation to each recommendation. In addition, each interviewee provided some barriers and facilitators in line with the recommendations that they had experience of. These are presented in Table 4 below and were grouped to provide the facilitators and barriers in Fig. 2.

**Table 4:** Table of key recommendation and implementation considerations

Category	Key recommendation	Implementation considerations from roundtables and redesign meeting	Implementation considerations from interviews	Implementation considerations from ACCESS team member meeting
Target population	Always consider underserved groups; socioeconomic disadvantage linked to inequalities across the board.	Difficult to know what the population should be. Intersectionality of under-served groups means more than one group needs to be considered when designing trials. Under-served groups by their very nature may not routinely access services where we recruit from e.g. primary care.	Assumptions from researchers can pose an issue. For example, the incorrect assumption that because you are an ethnic minority, you will experience socioeconomic disadvantage. There may be a selection bias going on, and those experiencing socioeconomic disadvantage and lower education/ literacy level may be routinely left out of research/ not approached by staff due to this. As a minimum you should aim to include ethnic groups in line with the national census.	Information about the population with a particular condition or disease is not readily available. To make changes to include people that are usually excluded will take longer and require more effort.
	Diverse PPI.	Lack of experience with diverse groups up to now; difficult to develop lasting relationships with communities for isolated research projects.	More preparation is needed for the meetings and communication. You cannot be parachuted into a setting, a community group for example, and expect people to volunteer themselves. You need to build trust. One interviewee reported using the INCLUDE Ethnicity Framework successfully to remove barriers and recruit the right proportions from different ethnicities. Some PPI contributors can be quite experienced in research so people with less research experience should also be consulted.	Using existing PPI members is likely to involve people with higher literacy skills; need to include people with lower literacy.
	Ensuring eligibility criteria do not exclude under-served groups (routinely missed groups = prisoners, learning difficulties, serious mental health).	Eligibility criteria are often linked to safety, especially in drug trials, or trials are focussed on a specific population that is dependent on the research question.	Some eligibility criteria can be difficult to identify, or misdiagnosed, for example most learning disabilities are not formally documented, making it hard to identify this group. Refugees with asylum status may be easy to engage with, but those that are undocumented and often highly vulnerable are less likely to engage with services.	Implicit criteria needs to be considered e.g., recruitment methods that rely on reading and responding to a letter or email, being able to attend a particular clinic may exclude some groups.
Recruitment settings	Choose sites from diverse areas.	It can be more work for trials team to add sites that are not ‘research ready’.	Even when trials have sites in areas of ethnic and socioeconomic diversity such as Bradford and Leeds, most participants seem to be middle class and white.	Need to acknowledge the impact of working with sites new to research – may take longer and more training and monitoring. Just going to a site does not necessarily mean the population taking part will be diverse, cannot be the only method used to improve inclusion.
	Have more than one method of recruitment to capture the required population.	Will need increased costs and staffing.	Research teams are stretched enough as it is, and sites/trials often do not have dedicated trial staff to do this. When using non-research staff to recruit, e.g. ward nurses, additional training in recruitment methods and trials is needed.	People often rely more on the consent conversations than on written information, which can be brought into training for recruiters.
	Moving recruitment into the community.	This depends on the research question, and not possible in some circumstances, e.g. If it is a surgical trial, you usually need to recruit the people listed for surgery. A lot of our research is NHS focussed, and so if people are not accessing NHS services, they will be missed.	Approaching people in a familiar environment helps increase trust. Just turning up in the community and trying to recruit people does not work; it takes time to build trust and may not fit into trial timelines. Community engagement efforts for clinical research, requires a long-term investment and may not have immediate outcomes. Constraints of certain trials e.g. discharge from hospital following a diagnosis of heart failure means by its nature, the patient would need to be recruited in an acute hospital setting.	Building relationships with the community is difficult but there are examples, and we need to share them in the trial community – example of Talking Trials project in Cardiff as a way of building two way relationships. Researchers could offer skills in research methods, evaluation and writing to help with bi—directional relationships.
Interventions	Use videos to explain the intervention.	A lot of our world is now on screen and in video format, and is almost expected.	Videos do not always work for those hard of hearing or with visual impairment. Voiceovers in different languages to explain the patient journey were well-received in a maternity trial.	CTUs do seem to be using videos for patient information and dissemination, but not necessarily to help with the intervention delivery – this is dependent on the intervention.
	Employ staff from underserved group to deliver intervention.	Dependent on the availability of staff.	We should not have to rely on specific individuals; this can be tokenistic and offensive.	Don’t just employ diverse staff—engage with community partners for wider engagement and co-production.
	Provide cultural (or similar) awareness training to staff.	It may be a challenge to get all staff to engage with this, and to complete it.	We shouldn’t be too reductionist about cultural competency and awareness—researchers/ those consenting should always take an empathetic, friendly approach irrespective of the patient’s culture.	Cultural humility is the idea that self-awareness, self-reflection and supportive interactions should always be practiced, and in this context, that the recognition of diversity and power imbalance are very important in patient care—can also be known as ‘institutional accountability’[22].
	Consider alternative routes for delivery, as different methods will exclude different people.	Need to consider digital poverty, rural participants and limited internet access, the two are interlinked.	Age may be a barrier to digital methods, and those from an older age group may be more reluctant and/or unable to engage in digital alternatives.	Trials need to test the same intervention and therefore researchers need to consider if the intervention changes when it is delivered by different routes.
Consent & communication	Use simple language (lay language and simple concepts) and videos.	Reluctance of researchers to challenge REC approved processes due to the fear of extending study timelines.	REC may not approve this because it may be difficult to include all the key information in a simple information sheet. The word ‘trial’ can be difficult to explain to some people outside research, and people often associate it with a trial in law, which can be associated with punishment	The national reading age is around 11—12 years old. If you explain to RECs that you are doing this to be more inclusive, and give the reasons, they are unlikely to say no. It can be difficult getting recruiting staff to use the videos in recruitment settings, particularly, for example, in the emergency department. The purpose of this is not necessarily to increase recruitment but to increase understanding, and support informed decision making.
	Initially provide shorter information sheets (layered information).	May encounter issues with REC for not including key information, but it is fine to include these in addition to usual information sheets to aid the consent process.	It may be difficult to include all the key information in a shortened information sheet.	Sponsors and organisations need to have certain information given to participants for liability reasons, and therefore some text cannot be removed.
	Employ staff from underserved group recruit.	Dependent on the availability of staff.	Considered valuable, though focus should be on people having interpersonal skills such as empathy, listening and communication. Where there is not a diverse workforce, researchers could work with community ambassadors.	Recruitment is often out of our hands as we sit in an organisation, though we could try to advertise more widely. We could work with community researchers or help to develop community researchers in the communities. There is separate workforce work being done in the NHS and the NIHR.
	Provide cultural (or similar) awareness training to staff.	Unless this is mandated, it is unlikely that the people who could most benefit from the training would do it.	An example of staff coming from different backgrounds and discussing their culture at a break time was given as a good way to share and learn from each other.	Focus should be on skills and competency, rather than just awareness. Additional training in recruitment methods and processes for specific groups, such as people lacking capacity to consent, or people with learning disabilities may be required, as being aware of it is not enough.
	Use translation and interpreters.	There was little experience of this in trials, with some reliance on NHS interpreters. Also consider additional support for e.g., people with learning or communication difficulties.	Experience that funding is not in place for translation or interpretation and that this is even stricter for commercial studies. Translation and interpretation is not standardised by the NIHR, and different CTUs use different providers. One example of working with local authority interpreters and a suggestion to ask community groups what they do in regard to interpretation, as they may have experience. Ensuring the translation is accurate can be difficult and costly, in some cases this can involve getting someone to ‘check’ a translation. Examples of making videos in different languages to explain the trial, or clinical trials in general. Interpretation is needed as well, a lot of people may not be able to read and therefore translation of documents is insufficient.	It is not clear how translations are actually being used in trials, or whether it is effective. If translation and cultural adaptation is required for the intervention, researchers need to consider if the intervention is the same. We can’t just translate a consent form or PIS, it has to be throughout the trial, including being able to report adverse events. It is difficult to know which languages to include, particularly in trials where this cannot be identified in advance of inviting a patient. There needs to genuine efforts made to provide translation and interpretation, so that it is not tokenistic.
Outcomes	Allow proxy completion where possible.	You want to hear the patient’s voice where possible, but proxy completion means people can still be included if they are unable to.	Self-completion of measures raises questions about the acceptable level of support needed and the boundary between self-completion and proxy assistance. You don’t want people to complete outcomes for participants unnecessarily and should always try to include the patient voice.	There are only a limited number of proxy measures appropriate for certain populations because they haven't been developed. Data collection should be around supporting the patient's voice where possible and where that's not possible then considering alternatives like proxy reported. In some trials you might collect both knowing that some people may lose the ability to complete self-report in the trial and then you have some comparison.
	Choose outcomes with simple language or translate them/ get interpreters.	Researchers tend to use outcomes that have been used in research before and are validated. These measures might not be validated in a different language. Certain terms about health and illness do not always exist in different languages.	This must be planned for in advance, as it is difficult to implement during a trial.	Involve patients in discussions and choices around outcomes.
	Focus on key outcomes with participants if burden is too high.	No barriers identified.	No barriers identified.	Field experts know some measures do not work and are being used as they were used in previous research. This can mean the secondary outcomes are more important to patients and so the outcomes to focus on should be discussed with PPI.
Follow-up	Arrange travel or upfront payment (not only reimburse).	Upfront payments are usually not possible, organisation payment systems are not fit for purpose.	Experience of not being reimbursed, reduces trust and people will not want to take part in research again.	Issues with payment through universities. There are alternatives such as setting up payment cards.
	Allow different methods of data collection.	Costs need to be included.	Staff can be stressed and under-resourced, and this could increase the workload for them.	Validated outcomes may have conditions around how they are presented. There needs to be some consideration of the different methods being comparable.
	Be flexible with times for clinic visits (out of hours).	Limited by the appointment systems and working hours of staff.	Research teams are stretched and often understaffed, so out of hours may not be an option.	Availability of staff out or hours can be an issue.
	Consider home visits and more support.	No barriers identified.	People are already doing this, and no specific issues were reported.	Home visits take longer and are more costly so needs to be resourced appropriately.
Analysis	Undertake sub-group analysis (even if not powered).	Sub-group analysis is rarely powered and the relevant data is not routinely collected.	Suggestion to over-recruit under-served groups and do sub-group analysis on different categories.	Issues with subgroup analysis: be careful about singling underserved groups out—is it about the treatment effect, or acceptability of treatment offer/plan? Consider if subgroup analysis is appropriate. This has been requested by other patient groups: where this is been requested, trial teams should explain where this is appropriate and what information it can provide. i.e. that it is unlikely findings will be powered and should be exploratory.
	Collect the relevant data.	Can be difficult to collect the correct data to measure socioeconomic status, there are several measures, and it is sensitive information.	Staff involved in recruitment reported that sometimes they do not have ethical approval to collect certain data. It is difficult to collect screening data (for those not recruited), as you cannot collect personal data prior to consent.	There are several measures used for socioeconomic status: E.g. Deprivation index, postcode, social group via occupation, housing, income, education levels. We try to collect the minimum amount of data needed– but we are using the data to assess generalisability.
Dissemination	Use videos and short communications.	Cost and resource at the end of a project might be limited.	Lack of identified staff to lead on this at the end of the study means it is not done.	CTUs do seem to be doing this now, usually through companies during, or shortly after, writing the results up.
	Translation.	Cost and resource at the end of a project might be limited.	No standardised way of doing this across CTUs/ NIHR, which can mean staff don’t know where to start. Additionally, there are often extra costs incurred that staff may not originally have considered e.g. having someone to proofread the original translation. This is not always offered in translation/ interpretation services.	There are some examples of translated dissemination videos.
	Involve diverse PPI in dissemination plans.	Cost and resource at the end of a project might be limited.	No barriers identified.	No barriers identified.
Other	Sub-studies should ensure they include representation from people from underserved groups.	No barriers identified.	Not discussed.	Same issues with recruitment as for the whole trial. It is difficult to know who to aim to include, particularly in smaller samples.
	Process evaluations should consider how they look at differences between underserved groups, may not be powered but can help hypothesis generation.	No barriers identified.	Groups are not homogenous, there are complexities within ethnic minority groups (and other under-served groups).	May have similar issues with recruitment (if applicable) as for the whole trial.
	Monitoring recruitment and retention by group throughout the trial.	Issues around data collection and data protection.	Restrictions on what kind of data you are collecting and analysing for the trial.	This should be done throughout a trial. If this shows that we aren’t recruiting a representative sample, what do we do about it? Should we raise this to funders? It may slow or halt recruitment. Having time to do this is a real issue.
	Specific interviews or questions to try to understand reasons for withdrawal and if this differs by underserved group.	Needs to be asked in the right way.	Patients from minorities may be reluctant to discuss their health in detail, particularly if it is a taboo subject e.g., prostate cancer.	The need for better data reporting around dropout. People have the right to withdraw without giving a reason and this needs to be respected.

### ACCESS team member input

Following interviews, we had a collaborator meeting to specifically discuss implementation issues based on the ACCESS team’s knowledge and experience. Table 4 presents the key findings on implementation from the roundtable and redesign meetings in relation to the key recommendations, from the interviews and from the ACCESS team member implementation discussion.

To present the guidance the ACCESS team agreed on an infographic to highlight the main recommendations and a full report of the findings.

## Phase 5: Development of the STEP UP guidance

### Findings complied into guidance document (April–May 2023)

KB combined all the ACCESS findings into a guidance document, starting with the key recommendations from the redesign meetings and incorporating the findings on implementation.

### Review of guidance (April 2023)

#### ACCESS team member input

The ACCESS team reviewed and commented on the first report, with the main feedback that it needed to be streamlined and more focussed to make it more accessible. They also reviewed all document drafts throughout the guidance development and provided resources and examples for the guidance.

#### Deep End PPI panel meeting

The Deep End PPI panel (https://sites.google.com/sheffield.ac.uk/dera/home/dera-ppi-group) is a diverse group of PPI contributors from GP catchments in the most deprived areas of Sheffield. KB attended a Deep End PPI panel to go through the guidance and discuss the panel’s feedback. The panel thought it was a useful and important piece of work. All panel members liked the recommendations, and the panel thought that ‘using simple language’ was the most important thing for researchers to work on. They expressed that a lot of the recommendations were obvious to them and wondered why trials were not being done that way already. The panel members were keen to hear the results and wanted to share the website and recommendations with other research teams they were working with.

### Developing the STEP UP guidance website and other materials (March-November 2023)

KB contacted a creative healthcare agency (COUCH Health) in March to discuss the possibility of working with them to create a website and other dissemination materials. We discussed the project, the findings and they proposed making a report, a website and an associated infographic. KB initially sent the project details, and following the ACCESS team member and PPI feedback, sent the full report of findings.

COUCH Health initially suggested names for the guidance that represented the final version of the guidance (as opposed to naming it after the ACCESS project), this was reviewed by the ACCESS team and ‘STEP UP’ (Strategies for Trialists to promote Equal Participation in clinical trials for Under-served Populations) was decided on. They then developed an initial outline, and two drafts of the report and an infographic, all of which were sent to the ACCESS team for review, as well as the mock up website. This was finalised and produced by the company in November 2023.

## Discussion

### The guidance

The guidance can be found online (http://step-up-clinical-trials.co.uk) and is split into 6 sections: Recruitment and setting, Stakeholder engagement, Communication, Flexibility, Researcher training and hiring strategies, and Data collection. There are specific recommendations for designing trials in each section, and includes comprehensive and practical advice for implementation. Clinical trials are embedded in multiple layers of context, and as such trialists should think about community level factors, and the setting of the trial or the included population which may limit the inclusion of some groups. Although our scoping review focussed on four under-served groups, more groups were included during the other stages of the project and each trial will need to consider the included trial population and the under-served groups relevant to them, which can vary across trials and settings. As noted in our discussions around implementation, this can be a difficult task as information about the population is not always readily available, but discussion with clinicians working in the area, and the use of routine data [23] are potential methods of identification.

Although this guidance was developed for a UK and Ireland setting, we think much of the considerations could translate to other countries and settings. We included input from experts and PPI contributors who come from other countries and/or are UK immigrants, but they would not necessarily know the clinical trial landscape in their home countries. Although we are unsure of the availability of funding to resource these recommendations, particularly in Low- or Middle- Income Countries (LMICs) settings, we do think trialists in other countries could use the recommendations as well. Whilst the barriers and solutions to improving inclusivity in clinical trials are likely to be context-specific, the underpinning principles in this guidance may be transferable to other international contexts. For example, determining the appropriate trial population, engaging the right stakeholders, enhancing communication, and reducing the overall burden on potential participants through flexible recruitment, data collection methods, and reimbursement would be beneficial for researchers globally. Additionally, providing relevant training on equality, diversity, and inclusion tailored to each country's context could help researchers address issues related to minority populations in their regions.

There are many wider systemic issues that affect the inclusion of under-served groups, including resource limitations and a lack of monitoring and reporting of diversity in trial populations. These complex issues require addressing at multiple levels. This practical guidance is intended for researchers when making decisions at the design stage of a trial.

### Using the guidance

We encourage trial teams to use this guidance to change the way they have been designing trials to increase the inclusivity of their trials. Relevant resources are listed in the guidance, and there is a section on the Trial Forge website around improving inclusivity in trials (https://www.trialforge.org/trial-forge-centre/diversity/). By using the guidance in the early stages of designing a trial, the trial can be costed appropriately, and protocols can incorporate the strategies that may help in the recruitment of under-served groups. Our identification of the implementation issues can help to plan and manage the strategies recommended. To make the recommendations more accessible for people designing trials, we have produced an infographic which is available on the website and included in supplementary material highlighting the key considerations. In addition to being used in the design of trials, these recommendations may need to be monitored and there may be a need for amendments during the management of a trial.

It is important to note that there are relationships between factors of recruitment, study engagement, and retention that may be at play, and efforts to improve recruitment may impact on engagement and retention which should be considered.

### Limitations

Although there was little methodological evidence identified in the scoping review, we have identified strategies/activities to support more inclusive trials through working with a wider range of members of trial teams and patients and the public. To determine their effectiveness, evaluation will be needed.

The funding for this project was limited and so we had to make some decisions around the best way to use this resource. In relation to the scoping review, the search was limited to one database (PubMed) and we focussed on methodological evidence from the UK and Ireland and focussed on four specific under-served groups. This search could have been widened to include other under-served groups, and evidence from outside the UK and Ireland, but we thought the searches would lead to too many papers to sift through in this project, and the aim was to produce guidance for researchers in the UK and Ireland.

We involved a wide range of stakeholders in the research but we did not ask for detailed background from our PPI contributors and therefore do not know if there was representation from, for example, gender minorities or LGBTQAI + individuals. From the information provided by contributors, we had representation from people with multiple chronic conditions, serious mental illness and mental health conditions. We did not seek to include experts from other areas that may have been useful, such as sociologists, political scientists or economists, though we did include patients and the public, clinicians, NHS research staff, trialists, trials methodologists, and an interdisciplinary group of academics with other sources of substantive expertise. Two of three chosen trials had representation from the original trial team. Although this may have led to us not knowing about specific implementation issues for one trial, this was a theoretical ‘redesign’ and implementation issues were readily discussed in each meeting.

### The future

During the project, we identified activities and strategies being used to support more inclusive trials, but these were not necessarily being shared widely. Reporting these approaches will help to diffuse good practice amongst trial teams. It would be helpful for trial teams to report on the implementation and effectiveness (i.e. do evaluations) of the strategies being used to try to improve inclusion of under-served groups, and CTUs may need to support site staff to do this. However, some of these recommendations may not lead to increased recruitment but will increase the level of understanding and improve the decision making for a wider range of potential participants, which could ultimately aid retention. This is an important ethical consideration and perhaps a moral obligation as well.

**Evaluating the recommended strategies.**

Several strategies recommended in the guidance can be evaluated through a ‘study within a trial’ (SWAT), and SWATs aiming to improve the recruitment or retention of under-served groups are a priority for the Trial Forge SWAT Network. There are existing SWATs in the SWAT repository (https://www.qub.ac.uk/sites/TheNorthernIrelandNetworkforTrialsMethodologyResearch/SWATSWARInformation/Repositories/SWATStore/) relevant to activities suggested in the guidance and could be adapted to focus on under-served groups and existing SWATs looking at the use of videos (e.g. SWAT 15) and translated videos (e.g., SWAT 205), that can be adapted for use in other trials.

QuinteT Recruitment Intervention (QRI) methods [24] aim to optimise recruitment and informed consent in randomised controlled trials (RCTs), and these could be adopted to make improvements to the recruitment of under-served groups.

Translation was considered an important topic for evaluation as CTUs are including costs for this at present, and we are not sure of the impact of this. The first step to this could be to survey CTUs on their current use of translation and interpretation, and assessing whether this improves recruitment of ethnic minorities.

Across the ACCESS project, there was a recommendation to simplify the language used, and to make sure staff communicate appropriately to improve trust, understanding, acceptability and recruitment, but there is a gap in research relating to ‘recruitment conversations’ in relation to under-served groups.

CTUs are introducing strategies to improve recruitment of under-served groups, and evaluations of these strategies need to be conducted to ensure we are making the right changes to trials, and ultimately widening the inclusion to trials.

## Conclusion

We created the STEP UP guidance to help trialists design trials that are more inclusive. The guidance was developed with ethnic minorities, older people, people with impaired capacity to consent, people experiencing socioeconomic disadvantage, people with physical and mental health conditions, and other under-served groups in mind, and we hope the recommendations (where possible) will be applied across all trials and populations, making trials more accessible to not only the groups we focused on, but to other under-served groups too.

Supplementary Information.

## Supplementary Information

Supplementary Material 1.

Supplementary Material 2.

## Data Availability

All data underlying the results, apart from the qualitative data from the interviews, are available as part of the article and no additional source data are required. Qualitative data not included in this article cannot be made openly available due to the risk of identification of the participants. Requests for transcript data maybe made to the corresponding author and considered in line with Data Protection.

## Abbreviations

ACCESS: A collaborative study to identify the activities needed to improve representation of under-served groups in trials and understand their implementation
CTU: Clinical Trials Unit
EDI: Equality, Diversity and Inclusion
GP: General Practitioner
INCLUDE: Innovations in Clinical Trial Design and Delivery for the Under-served
MRC-NIHR-TMRP: Medical Research Council – National Institute for Health and Care Research – Trial Methodology Research Partnership
NIHR: National Institute for Health and Care Research
NHS: National Health Service
PPI: Patient and Public Involvement
REC: Research Ethics Committee
ScHARR: School of Health and Related Research (now called SCHARR—Sheffield Centre for Health and Related Research)
STEP UP: Strategies for Trialists to promote Equal Participation in clinical trials for Under-served Populations
UKCRC: United Kingdom Clinical Research Collaboration
