# Supplementary material for: Time to STEP UP: methods and findings from the development of guidance to help researchers design inclusive clinical trials
Source: BMC Med Res Methodol. 2024 Oct 2;24:227. doi: 10.1186/s12874-024-02342-y (PMC11445965; doi:10.1186/s12874-024-02342-y)

## Appendices

Table 1. Contributors to ACCESS

| Phase | Attendees (total) |
| --- | --- |
| Roundtable 1 | 3x NHS research staff, senior trial manager, trial coordinator and 8x PPI contributors (13) |
| Roundtable 2 | ACCESS team member, 3x clinicians, R&D director, statistician, trial manager and 5x PPI contributors (12) |
| Roundtable 3 | ACCESS team member, 3x NHS research staff, 2x senior trial managers and 5x PPI contributors (11) |
| Roundtable 4 | 3x NHS research staff, trial manager, researcher, 7x PPI contributors (12) |
| Roundtable 5 | ACCESS team member, 2x NHS research staff, 2x researchers, 6x PPI contributors (11) |
| Redesign 1 | Consultant, research nurse, trial manager, 2x PPI contributors (5). |
| Redesign 2 | GP, CTU director, 10x PPI contributors (12); one person from original trial team attended. |
| Redesign 3 | 3x clinicians, 8xPPI contributors (11); one person from original trial team attended. |
| Interviews | 4x clinicians, 3x CTU staff, 5x researchers, 1x NHS research staff and 2 community experts (PPI). (15) |

Under-served groups represented by PPI (where provided by the contributor) = ethnic minority, experience of socioeconomic disadvantage, comorbidities, disability, people with mental health conditions, carer (for someone with impaired capacity), older people.

Figure 1 – Jamboards from the roundtable meetings

Meeting date: 3^rd^ March 2022


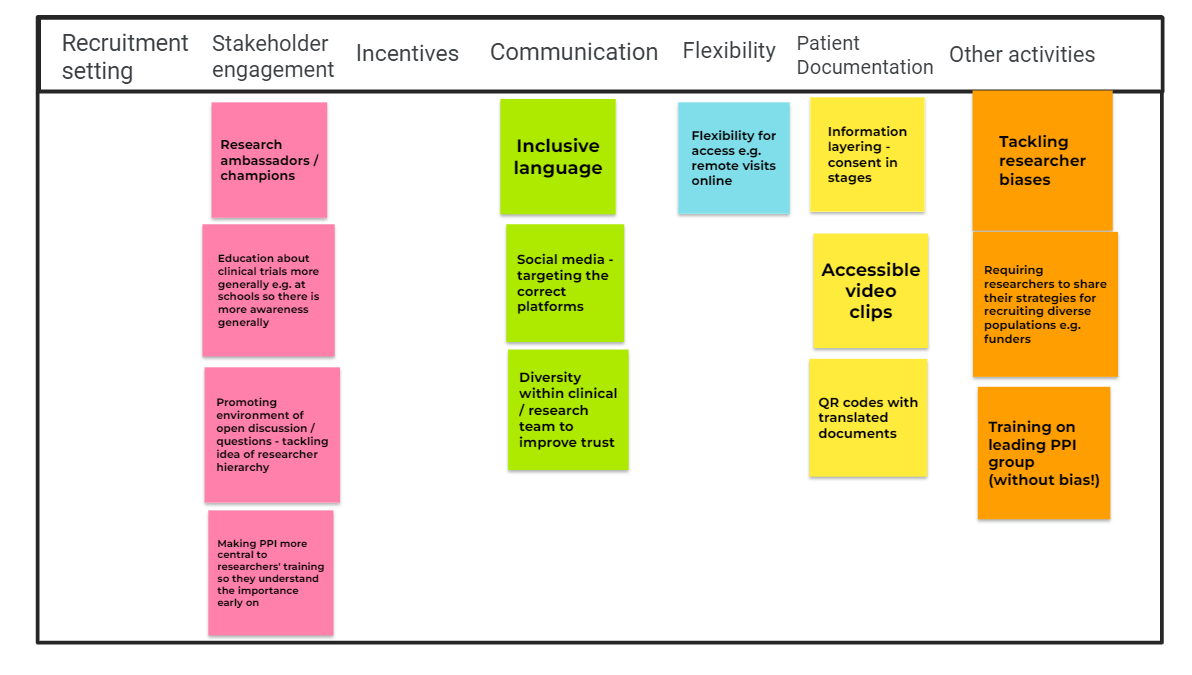


Meeting date: 7^th^ March 2022


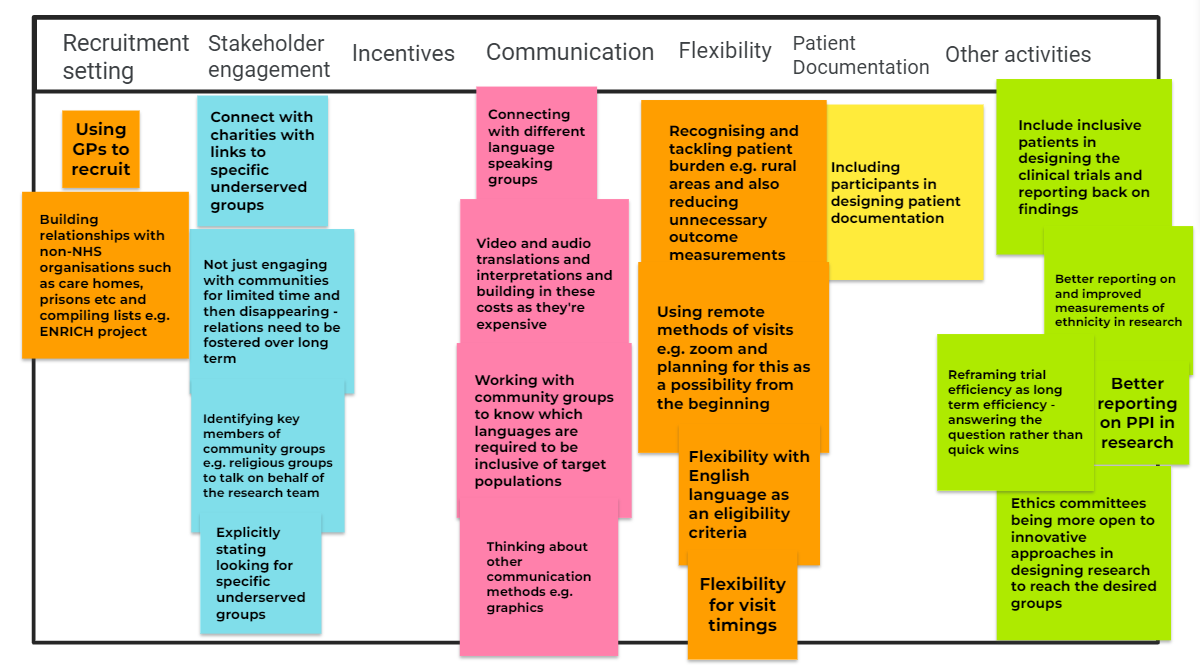


Meeting date: 18^th^ March 2022


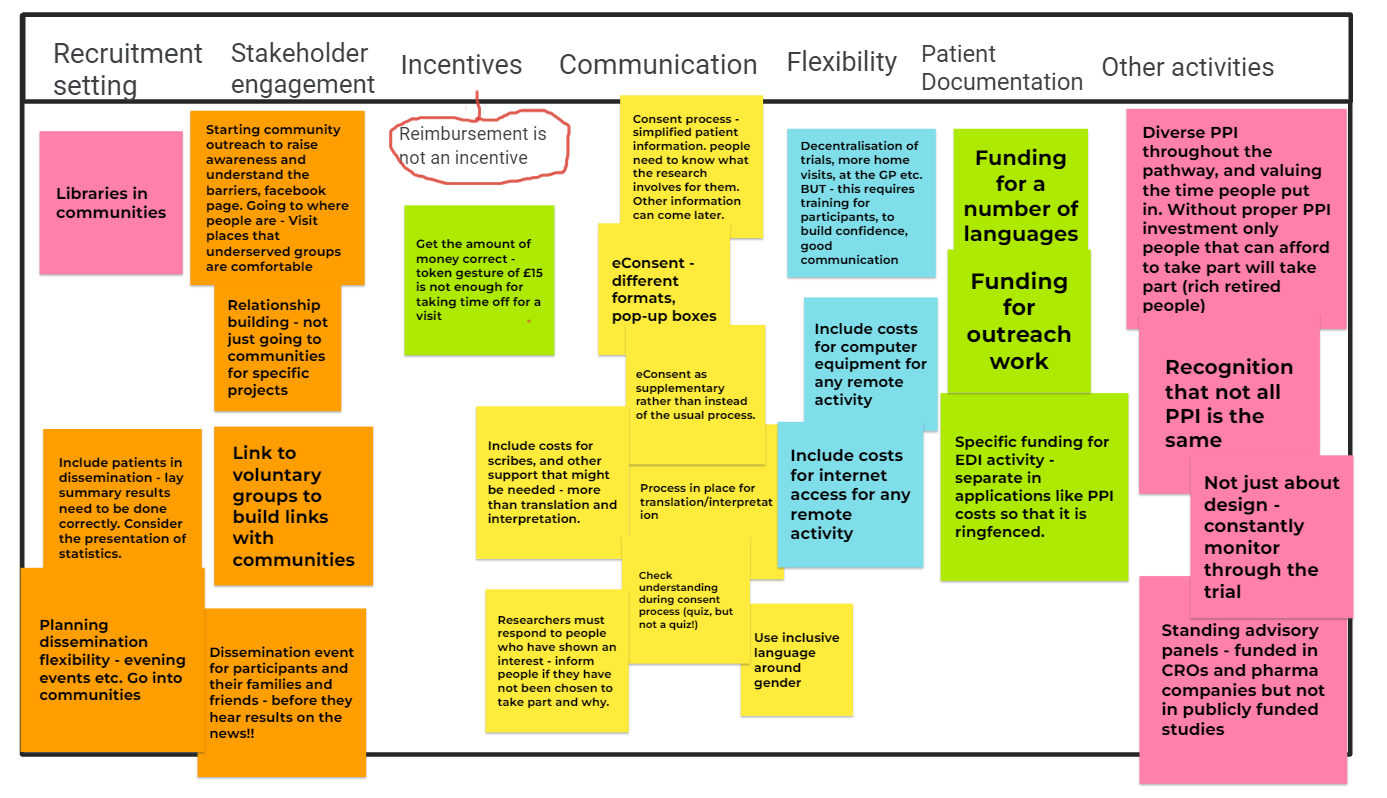


Meeting date: 25^th^ March 2022


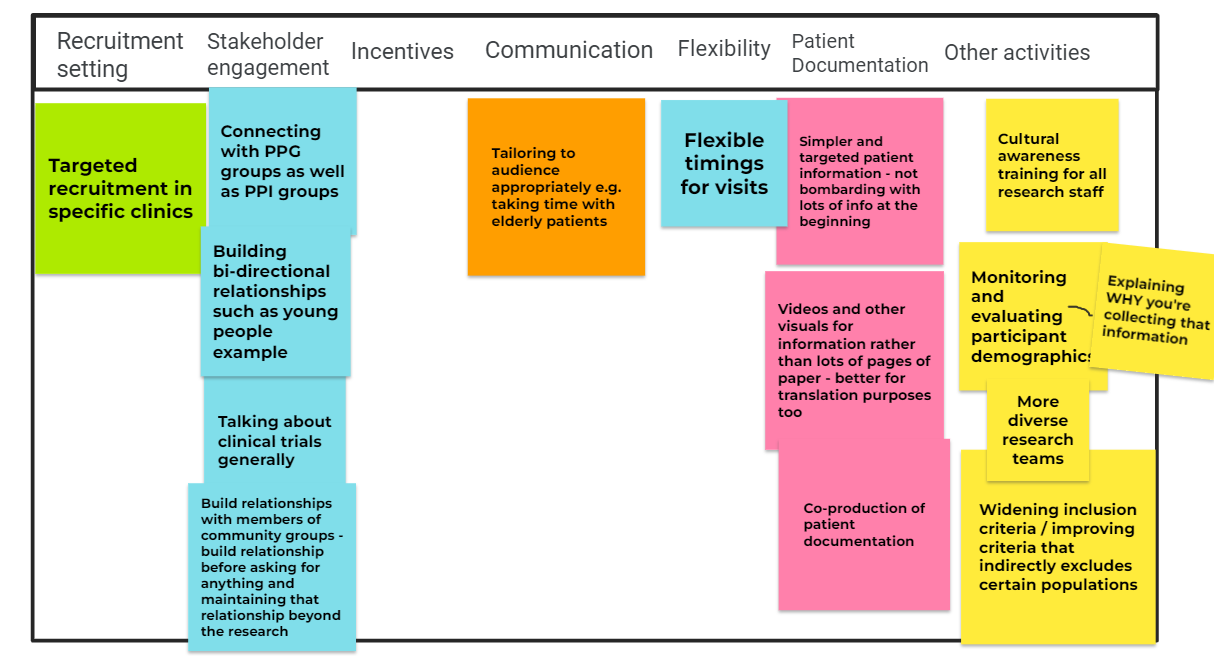


Meeting date: 28^th^ March 2022


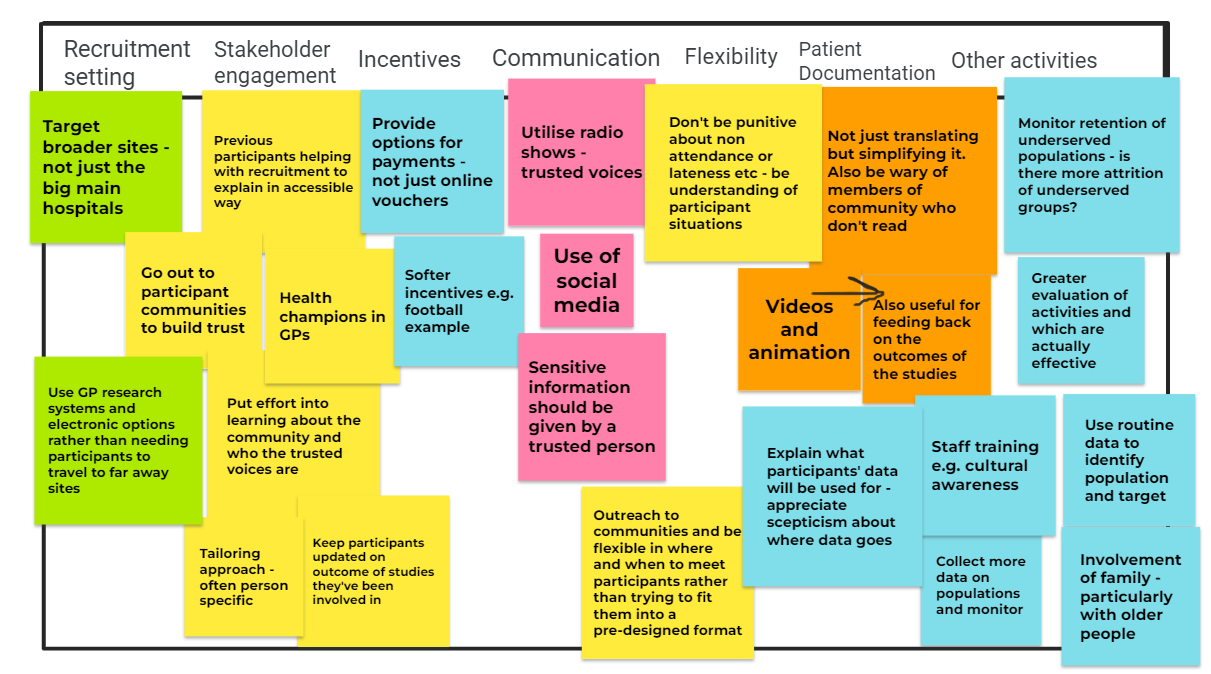

Supplement: Supplementary file 1 — Supplementary Material 1. [file 12874_2024_2342_MOESM1_ESM.docx]
