# Supplementary material for: Time to STEP UP: methods and findings from the development of guidance to help researchers design inclusive clinical trials
Source: BMC Med Res Methodol. 2024 Oct 2;24:227. doi: 10.1186/s12874-024-02342-y (PMC11445965; doi:10.1186/s12874-024-02342-y)
Supplement: Supplementary file 2 — Supplementary Material 2. [file 12874_2024_2342_MOESM2_ESM.pdf]

How trial teams can use STEP UP recommendations to support diversity in clinical trials

This infographic shows a summary of **STEP UP's** recommendations, designed to help improve the accessibility of clinical trials for groups under-served by research.

1 RECRUITMENT AND SETTING

Trial teams must accurately reflect patient populations in clinical trials.

They may choose to:

- Conduct equality impact assessments
- Include sites located in under-served areas
- Use baseline data to inform ongoing site selection
- Widen inclusion criteria where possible
- Use alternative recruitment pathways

How could implicit eligibility criteria, such as the expectation to speak English, impact diverse participation in trials?

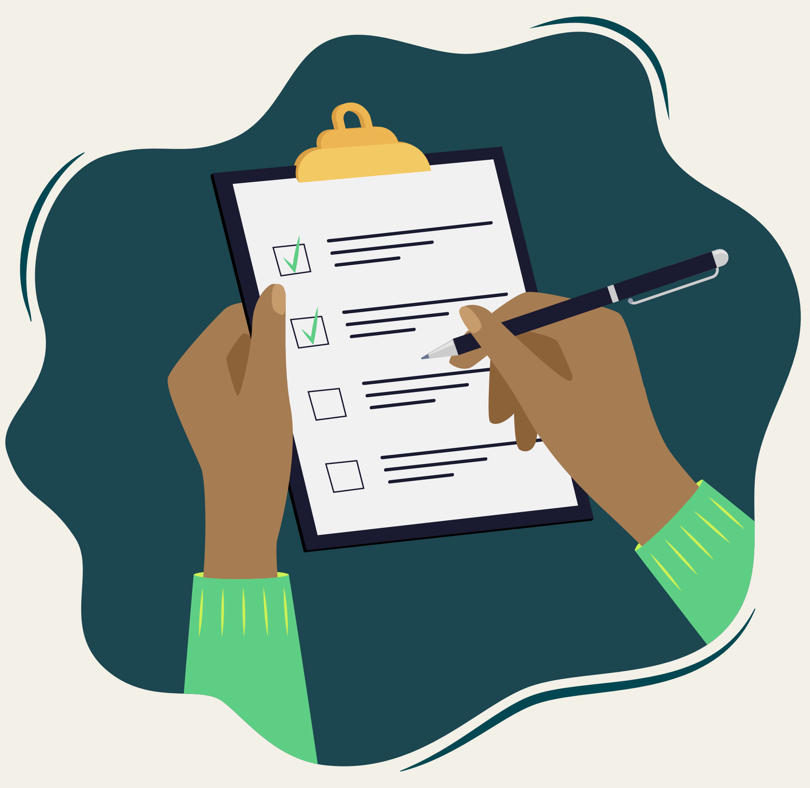

2 STAKEHOLDER ENGAGEMENT

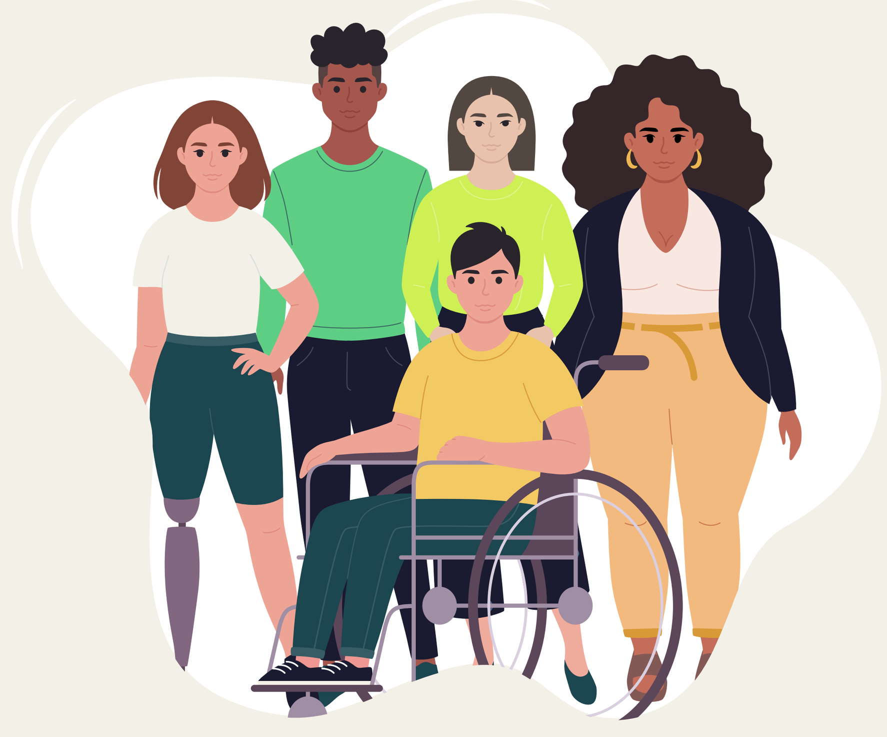

Working with people who have lived experiences of conditions is invaluable, but trial teams should also strive to engage with groups who are under-served by research.

They may choose to:

- Include the perspectives of under-served groups in patient and public involvement initiatives from the design stage
- Build relationships with communities under-served by research.

How can trial teams provide value to a community outside of a clinical trial?

3 COMMUNICATION

Trial teams should use effective communication to build trusting relationships with under-served groups and to reduce power balances.

They may choose to:

- Use simple, easy-to-understand language
- Tailor communications to the needs and preferences of different groups
- Use videos to supplement clinical trial materials in different languages if needed
- Ensure interpreters are available to translate throughout the trial
- Share trial outcomes with participants

Would the use of 'easy-read' materials help encourage greater participation from people with learning disabilities?

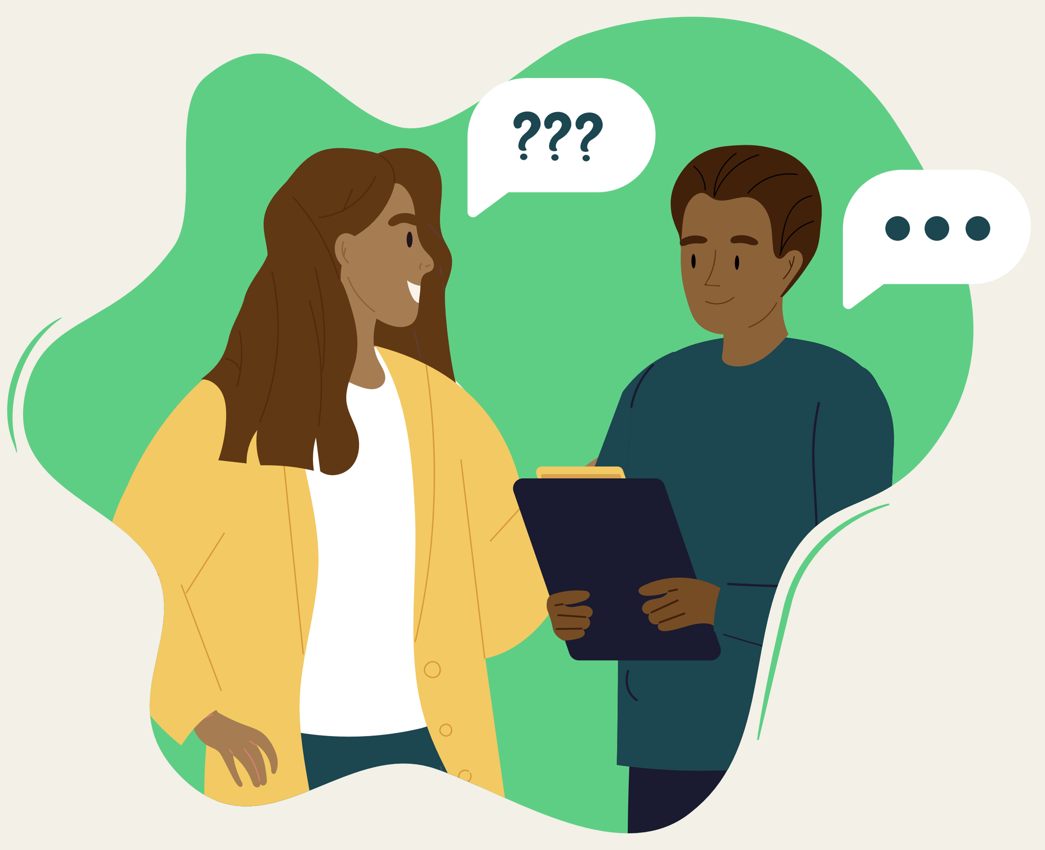

4 FLEXIBILITY

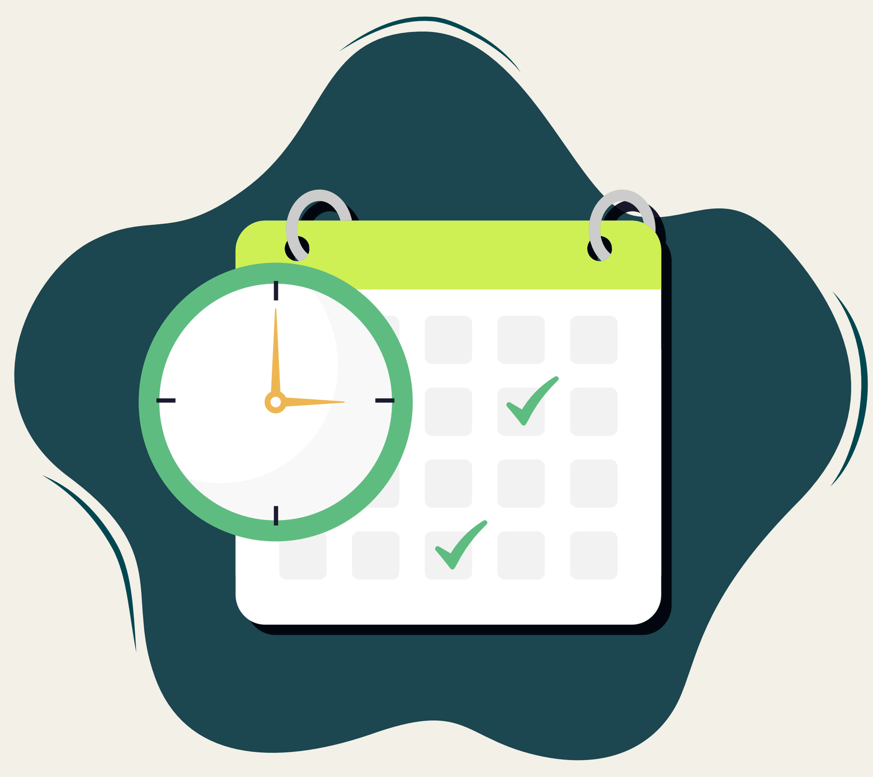

Trial teams should try to implement as much flexibility as possible throughout the trial.

They may choose to:

- Provide flexibility in recruitment methods
- Consider alternative delivery methods
- Be flexible with times for clinic visits
- Consider alternative incentives

Could the trial intervention be delivered more locally to the participant, such as in community venues or local GPs?

5 RESEARCHER TRAINING AND HIRING STRATEGIES

Trial teams should all have a thorough understanding of the impact of racism, prejudice, and ableism in healthcare.

Trial teams should:

- Be provided with cultural competency training
- Have a diverse range of backgrounds
- Be confident in communicating with patients who have disabilities

How can trial teams build on their knowledge of racism to reduce implicit bias?

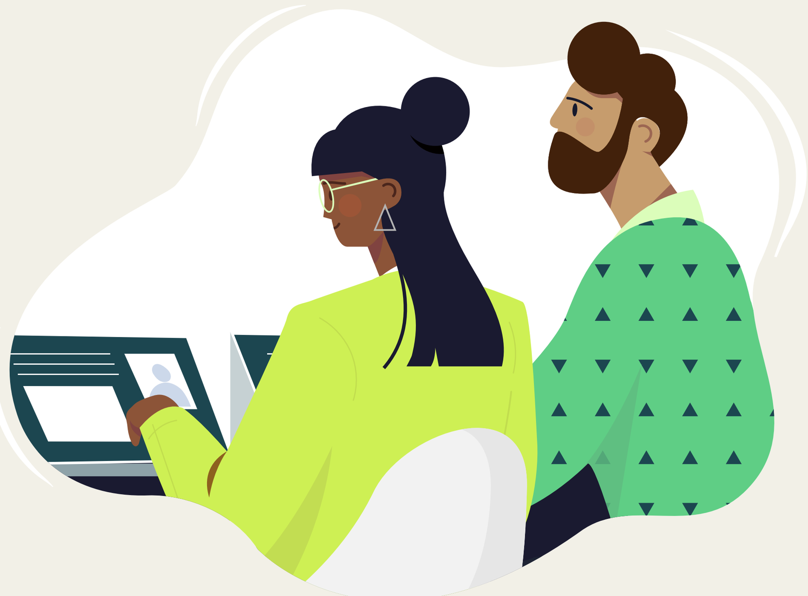

6 DATA COLLECTION

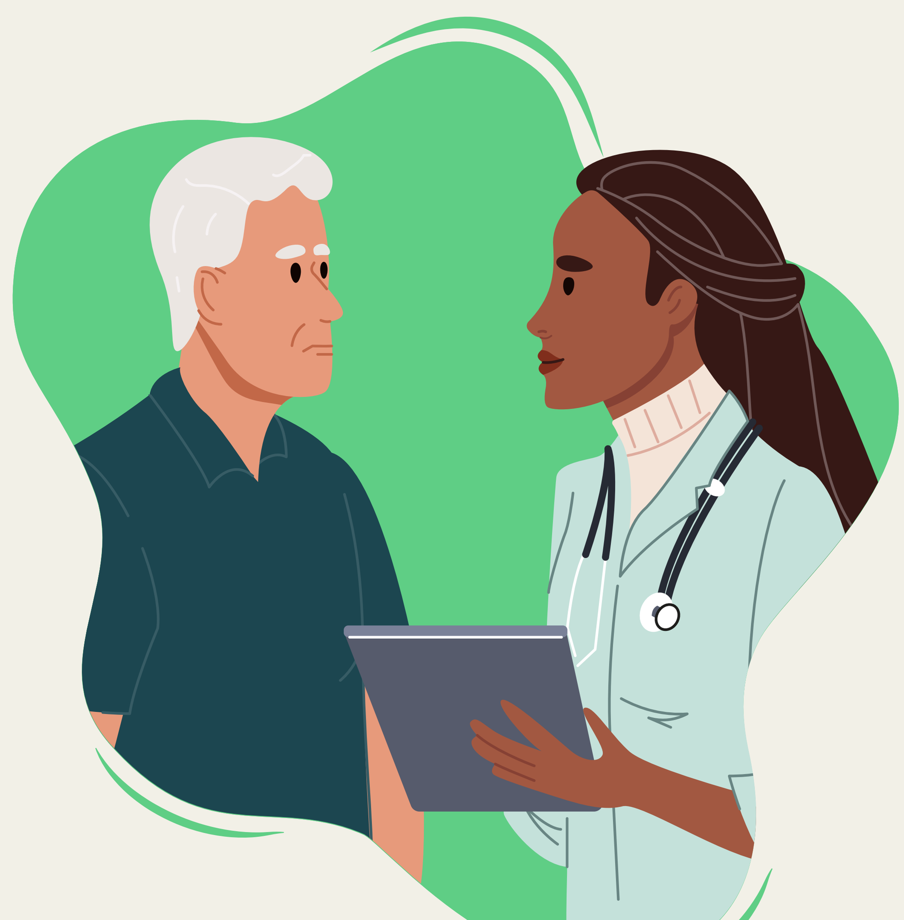

Trial teams should ensure data is collected and shared in a way that promotes flexibility and trust with under-served groups.

Trial teams may choose to:

- Collect demographic data of potential, lost, and current participants
- Identify trends to better understand why people from under-served groups drop out
- Allow different methods of data collection throughout the trial
- Allow for proxy completion to be carried out by friends or relatives
- Consider sub-group analysis

How can alternative methods of data collection enable people with disabilities to participate in clinical trials?

These recommendations are based on the findings of **STEP UP**. For more information about each recommendation, and to learn more about their considerations for implementation, please read the full report.
